# Free Dispensaries

**Published:** 1879-05

**Authors:** 


Free Dispensaries.—At the recent special meeting of the County Medical Society, held for the purpose of discussing the responsibility of the medical profession for the abuses of free medical services, one of the speakers related the case of an applicant at one of the dispensaries who demanded that he should receive attention before his regular turn, on the ground that he had a carriage waiting outside for him at the rate of a dollar an hour. Another told of a man, apparently in the most abject poverty, who was received as a free patient in the Manhattan Eye and Ear Hospital, and, while an inmate of the institution, died from an attack of pneumonia; yet when his clothing was examined after his death, the sum of fifteen hundred dollars was found in his pockets.—*Boston Medical & Surgical Journal.*

